# Digital interventions for substance use disorders in young people: rapid review

**DOI:** 10.1186/s13011-023-00518-1

**Published:** 2023-02-17

**Authors:** Marika Monarque, Judith Sabetti, Manuela Ferrari

**Affiliations:** 1 Douglas Mental Health University Institute, Montreal, QC Canada; 2 Department of Psychiatry, McGill University, Montreal, QC Canada; 3 Douglas Hospital Research Centre, 6875 Boulevard LaSalle, Perry C3 E-3102, QC H4H 1R3, Montreal, Canada

**Keywords:** Digital interventions, Substance use, Harm reduction, Young people, Mental health, Rapid review

## Abstract

**Background:**

Young people are disproportionately more likely than other age groups to use substances. The rise in substance use and related harms, including overdose, during the Covid-19 pandemic has created a critical need for more innovative and accessible substance use interventions. Digital interventions have shown effectiveness and can provide more engaging, less stigmatizing, and accessible interventions that meet the needs of young people. This review provides an overview of recent literature on the nature of recently published digital interventions for young people in terms of technologies used, substances targeted, intended outcomes and theoretical or therapeutic models employed.

**Methods:**

Rapid review methodology was used to identify and assess the literature on digital interventions for young people. An initial keyword search was conducted using MEDLINE the Cochrane Database of Systematic Reviews, Database of Abstracts of Reviews of Effects (DARE), Health Technology Assessment Database (HTA) and PROSPERO for the years 2015–2020, and later updated to December 2021. Following a title/abstract and full-text screening of articles, and consensus decision on study inclusion, the data extraction process proceeded using an extraction grid developed for the study. Data synthesis relied on an adapted conceptual framework by Stockings, et al. that involved a three-level treatment spectrum for youth substance use (prevention, early intervention, and treatment) for any type of substance.

**Results:**

In total, the review identified 43 articles describing 39 different digital interventions. Most were early interventions (*n* = 28), followed by prevention interventions (*n* = 6) and treatment interventions (*n* = 5). The identified digital technologies included web-based (*n* = 14), game-based (*n* = 10), mobile-based (*n* = 7), and computer-based (*n* = 5) technologies, and virtual reality (*n* = 3). Most interventions targeted alcohol use (*n* = 20) followed by tobacco/nicotine (*n* = 5), cannabis (*n* = 2), opioids (*n* = 2), ketamine (1) and multiple, or any substances (*n* = 9). Most interventions used a personalized or normative feedback approach and aimed to effect behaviour change as the intended outcome. Interestingly, a harm reduction approach guided only one of the 39 interventions.

**Conclusions:**

While web-based interventions represented the most common type of technology, more recently developed immersive and interactive technologies such as virtual reality and game-based interventions call for further exploration. Digital interventions focused mainly on alcohol use, reflecting less concern for tobacco, cannabis, co-occurring substance use, and illicit drug use. Specifically, the recent exacerbation in the opioid crisis throughout North American underlines the urgent need for more prevention-oriented digital interventions for opioid use. The uptake of digital interventions among youth also depends on the incorporation of harm reduction approaches.

**Supplementary Information:**

The online version contains supplementary material available at 10.1186/s13011-023-00518-1.

## Introduction

### Background

Adolescence and young adulthood are critical periods for first-time substance use, with peak levels occurring between ages 18–25 in most countries and for most types of drugs [1]. Alcohol use is most prevalent among young people worldwide, with 26.5% of 155 million adolescents ages 15–19 identified as users [2]: in Europe (43.8%), the Americas (38.2%) and the Western Pacific Region (37.9%) [2]. Concerning tobacco, 155 million people who smoke were identified in the 15–24 year age group for 2019, with an estimated global prevalence of 20.1% for males and 4.95% for females [2]. Smoking rates exceeded 33% for youth in the Pacific Islands, Europe (Bulgaria, Croatia, Latvia, France), Chile, Turkey, and Greenland [3]. Current use of the Electronic Nicotine Delivery Systems (ENDS) by youth (ages 8–20) was estimated at 7.8% [4], while past-month e-cigarette use among US teens increased 78% by 2017 [5]. Cannabis, considered relatively benign by youth when legalized [6], is the third most widely used substance [1]. Around 14 million or 5.7% of students 15–16 years old used cannabis in 2019 [2], with especially high use reported for Oceania (18%), the Americas (12.5%), and Europe (12%) [1]. Illicit drug use (heroin) among US high school students reached 7.0% in some urban centers by 2017 but was masked by lower national averages [7], while rates of cocaine, methamphetamine, and heroin use among young adults reached 11.4% in 2018 [8]. A US college study reported illicit substance use ranging from 6% for nonmedical use of prescription opioids to 21% for stimulants in 2020 [9].

Substance use in adolescence and young adulthood is associated with multiple adverse health outcomes, including high mortality (15–27%) among young people 15–29 years old from accidents and injuries due to alcohol consumption [2]. Alcohol and tobacco use were associated with increased long-term risks for cancers, cardiovascular and chronic respiratory diseases [10]. Health risks for young people who smoke included poor diet, inactivity, stress, and poor sleep hygiene, but also increased heavy episodic drinking, cannabis, and other drug use [11]. Studies observed the same progression to cigarette, marijuana, cannabis, and illicit drug use in vaping, as well as poisoning and severe withdrawal symptoms [5]. Research has identified marijuana use as a potential gateway to illicit drug use and the onset of psychiatric disorders in adolescents and young adults [12].

Self-isolation, social distancing and other public health measures enacted during the Covid-19 pandemic have exacerbated drug use and disrupted service delivery [13], creating barriers as unmet support needs increased among young people [14–16]. The global impact of the pandemic in terms of substance use and overall mental health has yet to be fully understood [17]. While increased tobacco and cannabis use was the single change noted in the early months of the pandemic across Europe [18], North American evidence showed overall increased substance use among young people [19, 20]. Trends also show associations between alcohol, tobacco and/or marijuana use and the initiation of illicit substances over time [9, 21–25]. In particular, the pandemic has worsened the ongoing opioid crisis [26], including misuse of prescription drugs by US youth [27]. The US and Canada reported more drug overdose-related deaths [28], with opioid toxicity deaths increasing roughly 66%, from 1,038 in 2019 to 1,792 by March 2021 [29].

The evidence on substance use interventions related to prevention, early intervention and treatment suggests that prevention interventions, typically delivered in educational settings, show greater effectiveness when targeting generic substance use than substance-specific programs [30] and may lower the odds of lifetime substance use [31]. Providing information on harms was ineffective, whereas skills development was a more effective approach [32]. Unfortunately, youth seeking substance use treatment have long faced multiple barriers related to treatment access, waitlists, costs, and stigma [33–35]. Moreover, many young people tend to delay or avoid help-seeking due to a preference for self-management [34], negative perceptions of services and professionals [36], and concerns about the stigma of mental illness [35, 37]. In fact, studies of young people in Western countries found that approximately 25% used services at all for mental health or substance-related problems [38–41], with many preferring the anonymity of online resources for accessing health information, education, and treatment [42–44]. As nearly all youth use the internet, and given the recent service environment, research on digital interventions has flourished, showing effectiveness for technologies based on internet, virtual reality, smartphones, video games, and telehealth for mental health problems [45–48], including substance use problems [32]. Digital technologies provide readily available, self-help alternatives and support for in-person treatment [15, 49, 50].

Few reviews concerned with digital interventions for youth substance use have been published [50, 51], with most focusing on a single substance (e.g., cannabis) or digital intervention (e.g. web-based intervention) without providing an overview of which digital interventions and technologies have been developed to support youth with substance use problems. Given recent trends in substance use and the shift to virtual treatment, this review provides an overview of recently published digital interventions with attention to how they meet user and research needs. This is the first rapid review to map the types of digital technologies in terms of substances targeted, level of treatment (prevention, early intervention, treatment) and expected outcomes, providing tangible information for researchers and front-line providers and with eventual relevance for young people using substances.

## Methods

### Research questions

The review addressed the following research question: What is the nature of digital technologies used in substance use interventions for young people, focusing on a single or multiple substances? The description and assessment of the various digital technologies included: (a) study and sample characteristics, (b) level on the spectrum of treatment interventions (prevention intervention, early intervention, treatment intervention) [52], c) targeted outcomes of the digital technologies for people using substances (e.g., behaviours, knowledge, perceptions of beliefs, attitudes, motivation or intentions); and (d) the underlying theoretical or therapeutic approaches used.

### Study design

Given the evolving shift to virtual care with the onset of the pandemic, rapid review methodology was used. While there is no consensus definition, the literature describes rapid review as a form of knowledge synthesis that streamlines and accelerates systematic review methods [53, 54]. The rapid review takes a more descriptive than critical approach and generally presents results as a narrative summary [55]. Easing certain requirements of full systematic reviews, rapid reviews may use a single research question, limit database sources and search years, and reduce research timeframes, allowing for timely completion and the delivery of recommendations to decision makers, healthcare professionals, policy makers or consumers, while saving resources [54, 55, 57]. The methods adopted in this study followed Khangura et al. [56]. The AMSTAR systematic review checklist is included in Supplementary Materials [57].

### Search strategy

The search strategy involved several databases: MEDLINE (via PubMed), the Cochrane Database of Systematic Reviews, Database of Abstracts of Reviews of Effects (DARE), Health Technology Assessment Database (HTA) and PROSPERO. The initial search was conducted in February 2021 for articles published between January 2015 and December 2020 and updated in January 2022 for studies published between January and December 2021. The search was limited to the previous 6 years, as research on digital interventions is a recent field. The terms used in the MeSH search strategy included: "Substance-Related Disorders" OR ‘’Smoking’’) AND ("Video Games" OR "Internet-Based Intervention" OR "Mobile Applications" OR "Virtual Reality" OR "Therapy, Computer-Assisted") AND ("Adolescent" OR "Young Adult"). Articles identified in the search were downloaded into the Endnote reference manager and duplicates removed.

The inclusion criteria were: (1) studies evaluating digital health interventions for substance-related disorders in youth; (2) primary, empirical studies using quantitative, qualitative and mixed-methodologies and review articles related to the topic; (3) populations including youth and young adults from 12 to 29 years old [58]. If a study age range was + or—1 year from our eligibility criteria, the study was included to ensure that important information was not omitted for the target population. For studies that expressed age as a mean rather than an age range, those with a mean age between 16–24 and a standard deviation below 4 were included; and (4) English or French language studies. Articles with interventions pertaining to substance-related disorders in combination with non-mental health issues (e.g., HIV or reproductive health) were excluded, as were dissertations and studies where participants were not exposed to the interventions (e.g., protocols, editorials, descriptive studies).

### Study selection and data extraction

Study selection involved a two-phase study identification process that included title/abstract and full text screening. First, two reviewers (MM and MF) independently screened titles and abstracts and assessed studies for inclusion using the Rayyan screening tool (Qatar Computing Research Institute), a web-based tool designed to facilitate study identification in knowledge synthesis projects [59]. Disagreements about study inclusion were resolved by consensus. Abstracts with insufficient information to screen for all the eligibility criteria passed directly to full-text screening. Second, for studies that met the inclusion criteria in the title-abstract selection, a single rater (MM) read the full texts to confirm that they met the inclusion criteria, and a second rater (MF) reviewed this work. Reviews and meta-analyses identified in the MeSH search were set aside for a secondary search of reference lists.

A data extraction grid was developed based on the following categories: (1) study characteristics: aims/hypotheses, setting, methods, targeted outcomes and main findings; (2) sample characteristics: age, population, sample size; (3) intervention characteristics: type of intervention (technology), substances targeted, description of intervention, theoretical or therapeutic model, and level or spectrum of the intervention (prevention intervention, early intervention, treatment intervention) [52]. As recommended by the Cochrane rapid review methods group, a single reviewer extracted the data (MM) and the others (MF and JS) verified the data for correctness and completeness [53].

### Data synthesis

The extracted data were synthesized, and interventions organized using an adaptation of a framework by Stockings et al. [52], which describes a three-level treatment spectrum for youth substance use interventions. 1) Prevention interventions aim to reduce interest in using substances, limit availability by making substances more difficult to obtain or consume, or discourage their use with criminal or other sanctions. 2) Early interventions identify youth at risk or showing signs of problematic substance use, aiming to reduce use before it escalates. They include harm reduction approaches focused on restricting or minimizing the negative effects of substance use. 3) Treatment aims at addressing problematic, heavy or dependent patterns of drug use and may focus on family, peers or the broader community as well as affected individuals ([32] p.282.). While Stockings also describes a number of important population-based interventions for reducing youth substance use, like restrictions on alcohol sales outlets, legal age limits on alcohol and tobacco use, and prohibitions against the use of controlled substances in many countries, these strategies were beyond the scope of this study which concerned only individual-level interventions. Targeted outcomes identified for each study were categorized according to behaviours, knowledge, perceptions or beliefs, attitudes, motivation or intentions, cravings, cognition, mood, skills, and functioning. Risk of bias assessments and critical appraisal of studies were not conducted due to the heterogeneity of study methodologies.

## Results

### Search results

**Fig. 1:**
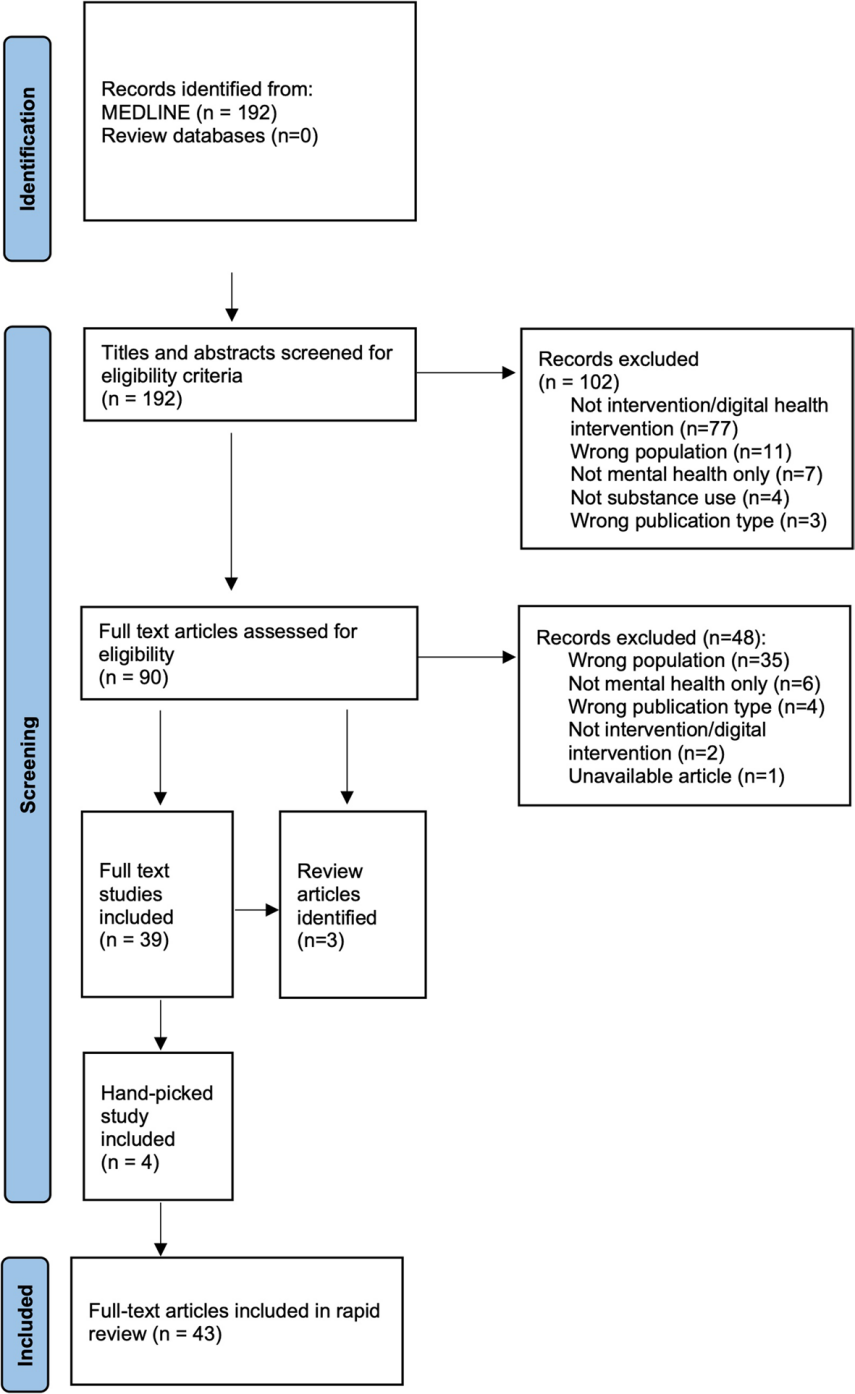
Study flow chart

The searches yielded 192 records, in total. Screening of the titles and abstracts for eligibility criteria produced 90 articles for full-text screening, with 102 records excluded. Of the 90 articles retained for full-text screening, 48 did not meet eligibility criteria and were excluded. A secondary search of reference lists was conducted on the three identified review articles [50, 51, 60], leaving 39 primary studies for review, to which 4 handpicked studies identified in the secondary searches were added. In all, the final review included 43 studies (See Fig. 1: Study Flow Chart).

### Study and sample characteristics

The 43 studies represent research conducted in 10 countries: the USA (25 studies), Australia (5), the UK and the Netherlands (3 each), Italy (2), and 1 each from China, France, Spain, Switzerland, and Taiwan. The studies described 39 different digital interventions that were distinguished in terms of which technology they used (See Table 1: Study characteristics and findings for 43 studies; See also Appendix 1: Definitions of digital intervention technologies). Just over one third were web-based (without a game component) (14/39: 35.9%), followed by game-based (10/39: 25.6%), mobile-based (7/39: 17.9%), computer or tablet-based (5/39: 12.8%), and virtual reality-based interventions (3/39: 7.7%). Web-based interventions were used in studies with youth and young adults ages 11–30, game-based interventions for ages 11–27, mobile-based (computer or tablet) for ages 12–35, and virtual reality interventions for ages 11–22.

**Table 1:** Study characteristics and findings for 43 studies

**Study**	**Substances targeted**	**Intervention name and description**	**Population/N/age (Mean or range)**	**Theory/Model/ Approach**^**a**^	**Method and main findings (re: substances only)**
**Web-based interventions**					
Bertholet, 2015 [61] Switzerland	Alcohol	Alcooquizz Elicited normative feedback on alcohol consumption and consequences per occasion; risk indication, alcohol and health info, recommendations	Young men (*n* = 737) with unhealthy alcohol use; mean age 20.75 (SD: 1.13)	Brief intervention; Personalized normative feedback	2 parallel-group RCT: -greater reduction in drinks/wk. for intervention group vs controls at 6 mo.; favorable intervention effect on AUDIT group vs. controls
Brunette, 2018 [62] USA	Tobacco	Let’s Talk about Smoking Brief, interactive, web-based, motivational intervention; info on smoking risks, exercises for self-efficacy, treatment, and treatment cessation; guided, 3-module program; personalized report	Young adults who smoke with severe mental illnesses (*n *= 81); mean age 24.8 (SD:3.6), range 18–30	Motivational decision support	Randomized pilot study: -more intervention users had biologically verified abstinence at 14 wks. vs. comparison subjects
Champion, 2015 [63]* Australia	Alcohol/ Cannabis	Climate Schools Alcohol and Cannabis course 12 lessons, 6/term offered at 6-month intervals; interactive online cartoons, teacher-delivered activities; info on minimizing harms of alcohol/cannabis use; aims to challenge perceptions of peer drug use, build resistance	Secondary school students (*n* = 1,103); mean age 13.25 (SD: 0.47), range 13–14	Social Influence; Harm Minimization approach	Cluster RCT: -sig. greater alcohol/cannabis knowledge at post-intervention, less alcohol consumption, and less intention to use alcohol in future for intervention group vs. controls
Newton, 2018 [64]* Australia	Alcohol /Cannabis	CAP (Climate and Preventure) program Two 90-min. group sessions delivered by clinical Psychologist; education; negative coping behaviors identified and challenged; coping and goal setting; analysis of physical, cognitive and behavioral responses	High school students (*n* = 1,712); mean age 13.3 (SD: 0.48)	Cognitive Behavioral approach; Psychoeducation	Cluster RCT; 3 intervention groups, 1 control: Universal Climate and combined programs increased cannabis-related knowledge up to 2 years; no sig. differences intervention vs. controls for cannabis harms and cannabis use
Teesson, 2020 [65]* Australia	Alcohol /Cannabis	Climate Schools Combined Intervention Six 40-min lessons on reducing anxiety and depression; same format as substance use course; incorporates skill acquisition, psychoeducation, management of psychological and cognitive symptoms and behaviors	Secondary school students (*n* = 6,386), mean age 13.5 (SD: 0.6), range 13–14	CBT; skills acquisition; Psychoeducation; Climate schools approach	Multicenter, cluster RCT: Combined intervention group increased knowledge re alcohol and cannabis, mental health; reduced odds of drinking, heavy episodic drinking, less increase in anxiety vs. controls
Davies, 2017 [66]** UK	Alcohol	Two interventions: “One too many”: a social embarrassment questionnaire and “Drinks Meter”: a personalized feedback app about personal drinking habits in relation to those of others	Young adults who drink currently (n = 488); mean age 21.70 (SD: 3.28), age range 18–30	Comparative behavior change techniques; Personalized normative feedback	RCT: Insufficient power to detect group differences
Deady, 2016[67] Australia	Alcohol	DEAL Project Automated, web-based self-help intervention for problematic alcohol use and depressive symptoms; 4 one-hour modules completed over 4 weeks; attention-control condition (Health Watch)	Young adults engaging in hazardous drinking (*n* = 104); age range 18–25	CBT, Motivational interviewing	Randomized trial: Sig. improvement on depression symptoms, symptom severity and reduced alcohol use, maintained at 6-mo. follow-up (H1 confirmed)
Geisner, 2015 [68] USA	Alcohol	Risky alcohol use intervention Brief, personalized, web-based feedback intervention for college students with both elevated alcohol use and depressed mood; customized programming with intervention material based on responses to screening and baseline surveys; info on referral	College students with AUDIT scores ≥ 8; and BDI-II score ≥ 14 (*n* = 311); mean age: 20.14 (SD: 1.34) range 18–24	Personalized normative feedback (Social norms approach); Psychoeducation; Skills/protective strategies	Randomized trial: No sig. main effects; moderation effects: participants in alcohol and integrated conditions with lower depressed mood or alcohol problems at baseline had greater reduction in alcohol-related problems at follow-up vs. controls
Marsch, 2021 [69] USA	Opioids	POP4Teens P4T: digital, interactive, activity-oriented program, unique in integrating knowledge about risk factors; education and skills training; JTT (Just Think Twice): active control condition	Adolescents (*n* = 406); age range 12–17	Psychoeducation; Social influence; Skills training	RCT: Sig. sustained improvements in intention to use prescription opioids (PO), increased perceived risk, and improved PO refusal skills for both programs. Sig. greater increased PO knowledge for P4T than for JTT
Miller, 2016 [70] USA	Alcohol	Personalized Feedback Interventions. 3 interventions: PFI-Norms group (Feedback on perceptions of peer drinking vs. actual patterns, and percentile rankings of drinking quantity on campus); PFI Enhanced group: same feedback + info on costs of alcohol consumption and behavioral strategies to limit risk); PFI-Choice group: choice of 2 (of 9) supplementary feedback components, also an assessment only (AO) group	College students, weekly alcohol use (*n* = 376); mean age 19.79 (SD: 2.17)	Personalized normative feedback	Exploratory RCT: The 3 PFI groups had greater decreases in alcohol use, peak blood and alcohol concentration (BAC), related problems and perceptions of typical students’ drinking vs. AO, but no differences among the PFI groups
Schuckit, 2015 [71] USA	Alcohol	Two sets of 4 educational videos, one set based on low LR to alcohol (Low LR-based (LRB) group) and the other incorporating more generic state-of-the-art (SOTA) information with no emphasis on model of risk; LRB included information on environmental mediators and attitudes, while SOTA focused on negative affect and impulsivity as additional risk factors. Drinking outcomes over 8 weeks were compared	College freshmen, past-month alcohol use (*n* = 454); age: NA	Motivational interviewing, Brief Intervention (with feedback)	Randomized trial: Both LR groups decreased drinking in both education groups, but more decrease in low LR group with the LRB educational protocol; high LR group demonstrated slightly better outcomes with SOTA protocol; small to medium effect sizes
Schwinn, 2018 [72]* USA	Any substance	RealTeen Intervention sessions guided by older, animated narrator; focus on goal setting, decision making, puberty, body image, coping, drug knowledge, refusal skills (two sessions), and a review	Girls, general population (*n* = 788); age range 11–15	Psychoeducation; Skills training; Goal setting; Social learning theory; Resiliency framework	Randomized trial: Intervention group smoked fewer cigarettes, less binge drinking and higher alcohol, cigarette, and marijuana refusal skills than controls, and less peer drug use vs. controls
Schwinn, 2019 [73]* USA	Any substance	See above	See above	See above	Randomized trial At 2-yr. follow-up, for intervention group less past-month cigarette, marijuana, and “other” drug use; lower peer drug use, increased scores on drug refusal skills vs. controls; at 3-yr. follow-up, for intervention group less past-mo. cigarette and e-cigarette use, lower peer drug use, increased drug refusal skills vs. controls
Tuliao, 2019 [74] USA	Alcohol	Brief online feedback intervention for risk of alcohol use. Intervention provides participants with their alcohol risk levels and offers info on professional help to those with problematic alcohol use	College students (*n* = 721); mean age 20.81 (SD: 2.49)	Brief feedback intervention	Mixed method study: Feedback group sig. less likely to view info on services vs. no-feedback group. Sig. feedback group x stigma interaction effect – those with average/high stigma re substance use scores less likely to view feedback information
Vargas-Martínez, 2019 [75] Spain	Alcohol	ALERTA ALCOHOL Feedback intervention, consisting of preventive messages and personalized information	High school students (*n* = 1,247); age range 15–19	I-Change Model; Feedback intervention	2-arm cluster RCT: Reduced binge drinking (BD) at 4-mo. follow-up; higher perceived HRQoL associated with reduced BD, controlling for several socio-demographic variables
Vogel, 2020 [76] USA	Tobacco	Put It Out Project Intervention included daily posts, image and text, and a question eliciting comments; live sessions on smoking cessation (’The Doctor is In’) with a commenting feature. Q/A format for info and support	Young adult who smoke (sexual/gender minorities) (*n *= 165); mean age 21.4 (SD: 2.3)	US clinical guidelines; Transtheoretical models of behavior change	Pilot RCT: POP more likely than TSP-SGM to report smoking abstinence at 3- and 6-mo. and reduced smoking at 3 mo.; reduced abstinence at 3 and 6 mo. and reduced smoking at 3 mo. for POP vs. those referred from Smokefree.gov
Walukevich-Dienst [77] USA	Cannabis	Personalized feedback intervention The PFI included PNF and feedback on: (1) risk related to cannabis use; (2) norms related to cannabis use and (3) risk for CUD	Undergraduate students with problematic cannabis use (*n* = 204); mean age 19.83 (SD: 1.43)	Personalized normative feedback	RCT: Gender moderated the relationship between condition and one-mo. follow-up problems, with fewer cannabis-related problems for women in PFI condition at follow-up vs. women in PNF-only condition. No sig. differences for men
**Game-based interventions**					
Abroms, 2015 [78] USA	Opioids	Recovery Warrior Two modes in game prototype with whole-body motion and voice recognition features. Players shout refusal phrases, e.g., “I’m clean!”; Object of game is to destroy drugs (‘’Recovery Ninja’’) or avoid drugs (‘’Recovery Runner’’)	Young adults in outpatient treatment (*n* = 9); age range 18–24	Social Cognitive Theory; Repetitive Priming; Reinforcement Theory of Motivation	Pilot survey: High satisfaction with video game over 4 weeks; all recommended playing the game weekly or more as part of treatment, a third recommended daily use
Boendermaker, 2015 [79]*** Netherlands	Alcohol	Cheese Ninja game 4 versions of training compared in terms of effects on motivation to train and on alcohol-related memory bias and alcohol use; original training, placebo, gamified and social versions of game compared; three training sessions, held 1–7 days apart	Undergraduate students drinking regularly (*n* = 77); mean age 22.7 (SD: 3.1), range 18–29	Cognitive Bias Modification	Non-randomized trial: No training effects but adding social elements to game enhanced user experience
Duncan, 2018 [80] USA	Alcohol and tobacco	*smoke-*SCREEN Video Gameplay 1 h, twice a week X 2 weeks; aim is to succeed academically and socially in 30 days of HW; avatar used to make decisions re earning good grades and social points in social situations involving cigarettes and marijuana that threatened success in both areas	Middle school students, naïve to cigarette/marijuana use (*n* = 25); age range 11–14	Behavioural skills development	Pretest-post-test design: Improved knowledge on both cigarette and marijuana uses from pre- to post-test, with medium-large effects. Positive reports on gameplay experience
Earle, 2018 [81] USA	Alcohol	CampusGANDR v2 Followed earlier research showing efficacy of personalized normative feedback (PNF) college alcohol interventions with added gamified elements (points, change, competition, personal avatars); this study tested a self-sustaining version of game, involving gameplay over 6 weeks (6 rounds); Players received PNF + reflective peer judgements of self-reported drinking behavior, norms	First year university students (*n* = 276); age: NA	Self-determination Theory; Personalized normative feedback	Non-randomized trial: Participants drinking heavily, who received both descriptive and reflective feedback on peer alcohol use, had sig. reduced normative perceptions and reduced alcohol use at 2-mo. post-intervention vs. those who received feedback on control topics
Hides, 2017 [82] Australia	Alcohol	Ray’s Night Out Intervention involves taking Ray, a red panda avatar, on a ‘relaxed’ ‘fun’ or ‘crazy’ virtual night out; aim is to provide users with information, motivation, and behavioral skills to set a drinking goal for the night, keeping Ray below his ‘stupid line’ for drinking	Young people, alcohol use in past mo. (*n* = 197); age range 16–25	Motivational Interviewing; Information-Motivation-Behavioural skills health behaviour model; Social Learning Theory	RCT: Immediate access group had sig. greater increase in alcohol knowledge than delayed access group at 1-mo.; no group differences in alcohol use; for both groups, sig. reduction in number of drinks per occasion and in maximum number at 1 mo. and in alcohol-related harms
Jander, 2016 [83] Netherlands	Alcohol	Alcohol Alert Game includes an online baseline questionnaire; adolescents then play 3 sessions of the 2-dimensional game “What happened?!”; tailored to typical consumption in different situations; questions and feedback; action plans provided; 4^th^ session outside of game and follow-up questionnaire after 4 months	Secondary and vocational students (*n *= 2,649); mean age 16.3 (SD: 1.2), range 15–19	I-Change model; Feedback intervention	Cluster RCT: Intervention reduced binge drinking among adolescents and those 16 years + after at least 2 intervention sessions; prolonged use of game was associated with stronger effects for binge drinking
LaBrie, 2019 [84] USA	Alcohol	Gamified PNF with virtual co-presence; level manipulated across 3 conditions, all gamified (PNF Only, PNF + Visual Copresence, and PNF + Maximum Copresence), plus non-gamified PNF control. Same questions about drinking asked and identical PNF delivered on alcohol use but different levels of visual and text-based information about peers from other universities	Undergraduate students reporting consumption of at least one alcoholic drink in previous 2 weeks (*n* = 235); age: NA	Personalized normative feedback	Randomized trial: Drinking sig. reduced only in gamified condition with maximum copresence; outcomes relative to standard PNF. Both gamified conditions with copresence sig. improved upon standard PNF in reducing alcohol use at follow-up among people with heavier alcohol use heavier pre-intervention
Sanchez, 2015 [85] USA	Any substance	Arise Instructional segments and skill-building games teaching coping skills: relaxation lesson; “Blown away game’’ for diaphragmatic breathing; a lesson on 5 refusal techniques and the ‘’stand up game’’, aimed at selecting an appropriate refusal from several options	Adolescents in substance abuse treatment (*n* = 9); age range 15–17	Coping skills training	Quantitative descriptive: High overall usability, acceptability, and utility ratings by adolescents and providers in pilot study. Both relaxation and refusal units were highly rated, but game components ‘’Blown away’’ and ‘’Stand up’’ were rated lower than other components
Scholten, 2019 [86] Netherlands	Tobacco	*HitnRun* Social mobile or ‘’runner’’ game, where players control an avatar who runs forward and collects points; tailored prompts reminding users with high levels of craving to play the game; 4-member teams encouraged gameplay, with a bonus if everyone played	People who smoke motivated to quit (*n* = 144); age range 16–27	Peer contagion; Go/No-Go training	Two-arm RCT: Similar reductions in weekly smoking levels and abstinence rates for both groups; dose effect with *HitnRun* only: lower weekly smoking levels with longer gameplay; for brochure group, higher dose related to higher weekly smoking levels throughout study
Skorka-Brown, 2015 [87] UK	Alcohol, nicotine, caffeine	Tetris Questionnaire (both groups) + ecological momentary assessment with SMS messages on iPods to prompt craving assessment; intervention group also played Tetris for 3-min. and reported cravings again	Undergraduate psychology students (*n* = 31); age range 18–27	Elaborated Intrusion Theory; Visual Interference	Randomized trial: Playing Tetris decreased craving strength for drugs (alcohol, nicotine, caffeine), food and other activities (sex, gaming, exercise), consistent effect across the week
**Mobile-based interventions**					
Boendermaker 2015 [79] *** Netherlands	Alcohol	Alcohol Go/No-Go Training Training to avoid automatic motivational approach tendencies toward alcohol using a mobile application. Motivation and user experience, approach bias, alcohol problems and use, compared to a standard computerized version. Follow-up email questionnaire 2 weeks later	University students- drinking regularly (n = 64); mean age 22.44 (SD: 2.58), range 18–35	Cognitive bias modification	Pilot study No training effects
Carrà, 2015 [88]* Italy	Alcohol	D-ARIANNA Questionnaire on binge drinking behavior and factors contributing to overall risk used to develop a risk estimation model for binge drinking that was incorporated into the D-ARIANNA health app	Adolescents, young adults (*n* = 110); age range 16–24	Risk Estimation Model	Lit review and development of risk estimation model: Ten risk factors (5 modifiable) and 2 protective factors were sig. associated with binge drinking and included in the model. Most participants (73%) regarded the eHealth app as helpful to assess binge drinking
Carrà, 2016 [89]* Italy	Alcohol	D-ARIANNA E-health app with questionnaire, identifying risk/protective factors were entered into an algorithm and based on estimation model identified low, moderate and high-risk models for individual participants	Young adults (*n* = 590); age range 18–24	See above	Quasi-experimental, pre-post study: Diminished BD shown at follow-up and confirmed in an appropriate generalized estimating equation model with unweighted data on a last observation carried forward basis
Coughlin, 2021 [90] USA	Alcohol and cannabis	MiSARA Smartphone app designed to reduce substance use; 30-day intervention included daily and weekly surveys, tasks, with inspirational messages and reminders; groups randomized to receive tailored message, fun fact on random topic or no message	Youth who binge drank or used cannabis in the past month (n = 39); age range 16–24	Personalized feedback; motivational interviewing; mindfulness; behavioral activation; support based	Qualitative study: Most (79%) liked the app. but more interactivity wanted. Substance use declined over time; more frequent users of app reported less substance use at 1-month follow-up than others
Davies, 2017 [66]** UK	Alcohol	Drinks Meter Drinks Meter smart phone and online digital app, offers personalized feedback on drinking as compared with peer drinking; info on calories consumed and money spent relative to others; risk assessed and advice on how to reduce consumption	Young adults, self-identified drinkers (*n* = 488); mean age 21.70 (SD 3.28), range 18–30	Personalized normative feedback; Psychoeducation	See outcomes above (Davies, 2017)
Dennis, 2015 [91] USA	Any substance	ACHESS Intervention randomly prompted participants with Ecological Momentary Assessments 6 times a day. They could then access ecological momentary interventions including recovery support and motivation, relaxation and social networking	Adolescents at discharge from residential treatment (*n* = 29); age range 14–18	Ecological Momentary Assessment and Intervention	Non-randomized trial Sig. higher rates of use for “unrecognized risk” and “current use” groups vs. recognized risk group over a week. Sig. lower use in subsequent week when EMI accessed 2 + times within the hour following an EMA vs. when EMIs not accessed
Haug, 2017 [92] UK	Alcohol and tobacco	*MobileCoach Tobacco* (MCT) vs. expanded *MCT* + Implemented original MCT program targeting smoking cessation only with expanded program (MCT +) that integrated smoking cessation and alcohol reduction	Vocational students who smoke (*n* = 1,471); mean age 18.6 (SD 3.1)	Health Action Process Approach (HAPA); Personalized normative feedback (Social norms approach); Social Cognitive Approach, mindfulness, behavioural activation	2-arm, parallel-group cluster RCT: No significant group differences observed for either primary or secondary outcomes
Kazemi, 2019 [93] USA	Alcohol	*SmarTrek* SmarTrek addresses alcohol use through eight functions: e.g., tracking features for drinking behavior, a virtual coach, daily text messages and alerts, strategies and feedback for changing drinking behaviors, education, and links to local resources	Undergraduate students, past month alcohol consumption (*n* = 10); Mean age 22.7 (SD: 7.66)	Motivational interviewing; Ecological Momentary Intervention; Personalized normative feedback	Mixed methods: Theater testing, field testing and focus groups found that the *SmarTrek* app was easy to use, information was useful and had a positive effect on decreasing their drinking
**Computer or tablet-based interventions**					
Ellis, 2017 [94] USA	Alcohol	Computer delivered brief intervention (CDBI) Included 3 components; (1) decisional balance: reported likes/dislikes about alcohol use; (2) normed feedback: info given on drinking compared with others same age and gender; and (3) goal setting aimed at behavior change. Tailored responses based on participant drinking behaviors	Undergraduate students (*n* = 103); age: NA	Brief feedback intervention; Motivational interviewing	2-group experimental study: High empathy brief intervention participants had increased motivation to reduce drinking, felt more supported and less criticized vs. low empathy condition
Jacobus, 2018 [95]* USA	Cannabis	Cannabis Approach Avoidance Training (CAAT) Computerized CBM focused on impulse to approach rather than avoid a substance cue. The image format directs individual to push a joystick when substance cue is presented, and pull it when nonsubstance cue is presented, thereby training the participant to “avoid” the targeted substance cue	Students using cannabis weekly not seeking treatment (*n* = 80); mean age: 19, range 17–21	Cognitive Bias Modification	Mixed models repeated measures analysis: Sig. group x time interaction effects predicted percent days of cannabis and alcohol use over study enrollment period. For CAAT group, 7% fewer days of cannabis use vs. 0% for controls; for avoid cannabis condition, 10% more alcohol use days vs. 3% more for controls
Karoly, 2019 [96]* USA	Cannabis	Cannabis Approach Avoidance Training (CAAT) + pre-post MRI Six sessions of CAAT training (or CAAT sham – control) twice/wk. over 3 weeks; baseline and post-treatment visit included a 30-min. MRS scan, including a visual cannabis cue-reactivity task	Youth regularly using cannabis (*n* = 37); age range 17–21	Cognitive Bias Modification	Pre-post intervention: Group-time interaction for CAAT vs. CAAT-sham reached trend-level significance. Change in approach bias sloped from pre-post treatment was positive for CAAT-sham (increased approach bias) and negative for CAAT training (change to avoidance bias)
Knight, 2019 [97] USA	Alcohol and tobacco	Computer-Based Substance Use Screening and Brief Behavioral Counseling Self-administered screening and brief intervention with immediate feedback on CRAFFT score and level of risk; psychoeducation includes 10 interactive pages of scientific information and true-life vignettes illustrating the health risks of substance use; motivational interviewing offered by practitioners	Youth using cannabis for 23-mo. (*n* = 965); age range 12–18	Motivational interviewing; Psychoeducation; Brief feedback	Intent to treat RCT: Adjusted hazards ratios for time to first post-visit use of alcohol or other drugs for CSVI vs. US: alcohol use (0.69), heavy episodic drinking (0.66), and cannabis use [61]
Tello, 2018 [98] France	Alcohol	Evaluative Conditioning Brief intervention The intervention consisted of repeatedly pairing a word (related to alcohol, soft drink, or neutral) with an image (positive, negative, or neutral). In the evaluative condition (EC), a negative picture followed the words related to alcohol, a positive picture after words related to soft drinks and neutral picture following neutral words, vs. neutral or positive pictures in control condition	Second year university students (*n* = 122); mean age: 19.84 (SD: 2.02)	Cognitive Bias Modification	Pre-post intervention study: Evaluative conditioning (EC) did not change the implicit evaluation of alcohol but did reduce drinking behavior. This effect was independent of hazardous drinking behavior, but was especially pronounced among participants with the most positive implicit evaluation of alcohol before the intervention
Walton, 2015 [99] USA	Alcohol	Brief alcohol interventions Three conditions included: a Therapist Brief Intervention (TBI), Computer Brief intervention and an enhanced usual care control. The TBI used a computerized workbook with tailored feedback, with screens containing prompts to structure the session. The CBI was an offline Facebook-style program for tablet computers, with sections on normative feedback, personal strengths, and better things to do. The order of completion was optional	ED patients with positive screens for risky drinking (*n* = 836); age range 14–20	Motivational Interviewing; Cognitive Behavioral Therapy; Self-determination Theory; Personalized normative feedback	Pre-post-test survey: Sig. post-test increase for TBI on “importance to cut down” and “readiness to stop” and for CBI in “importance and likelihood to cut-down”. BI components positively associated with post-test outcomes: identification of personal strengths, protective behavioral strategies, benefits of change, and alternative activities (sports). Providing info during the TBI was negatively associated with post-test outcomes
**Virtual reality (VR)-based interventions**					
Guo, 2021 [100] Taiwan	Tobacco	Virtual Reality Game in Smoking-Prevention Education The educational VR games included a whack-a-mole game, a wire loop game, a square baseball game, and a Taiko drum game. Participating students wore a head-mounted display (HMD), enabling them to have immersive experiences with 3D images. For challenge tasks, participants interacted with the virtual environments using joysticks; they received in-game guidance from an avatar	High school students (*n* = 130); Mean age: 16.64	Keller’s ARCS (attention, relevance, confidence, and satisfaction) motivation model	Prospective observational study: Sig. improvement in knowledge; most students perceived themselves as persuaded to abstain from smoking and were sig. influenced by attention, relevance, and satisfaction
Man, 2018 [101] China	Ketamine	Virtual reality-based vocational training system Intervention for cognitive and vocational enhancement. The VR group used a 3D non-immersive virtual reality-based vocational training system (VRVTS) to create a virtual boutique. A second program (TAG) had similar content but was administered by a tutor, using the programme manual. The program included three levels, five sessions each: pre-trainee level, trainee level and sales level. To enter the sales level, participants had to complete tests for advanced attention, memory and problem solving	People using ketamine (*n* = 90); mean age 22.80 (SD: 5.41)	Cognitive and vocational skills training	RCT: Sig. improvement in attention and memory for VRG, maintained at 3-mo. follow-up; both VRG and TAG showed improved vocational skills after training, maintained at follow-up, and improved self-efficacy
Weser, 2021 [102]* USA	Tobacco/ Nicotine	Invite Only VR A Vaping prevention game, that teaches about health risks of vaping e-cigarettes and provides a virtual environment for adolescents to practice refusing vaping of e-cigarettes, Player uses four abilities to resist peer pressure: (1) observation of the environment, (2) ability to apply new knowledge about vaping in conversations, (3) deciphering vaping colloquialisms and (4) ability to refuse peers effectively	Adolescent students (*n* = 47); age range 13–15	Behavior change theories, including theory of planned behavior and Social Cognitive Theory	2-group pre-post intervention study: Pre-post increase in player knowledge, and perceptions of e-cigarette harm; decreased likelihood of future e-cigarette use. Game enjoyment and willingness to recommend the game were high
Weser, 2021a [103]* USA	Tobacco/ Nicotine	See above	Middle School students (*n* = 287); mean age 12.45, range 11–14	See above	Non-equivalent control group study: Sig. results from baseline to 6 mo. for intervention group on e-cigarette knowledge, nicotine addiction knowledge, perceived addictiveness of e-cigarettes, perceptions of harm, and social perceptions about e-cigarette use, vs. controls. High ratings on gameplay and VR experience and satisfaction,

^*^Intervention presented in study was evaluated in multiple studies

^**^Two different digital technologies evaluated in one study

^***^Two different digital technologies evaluated in two studies, within one publication

Just over half of the digital interventions targeted alcohol use (20/39: 51.3%), followed by tobacco or nicotine interventions (5/39: 12.8%), of which slightly more than half (3/5) addressed e-cigarette use. Digital interventions for cannabis use (2/39: 5.1%), opioids (2/39: 5.1%), and ketamine (1/39: 2.6%) were less common. Nine of the 39 interventions targeted multiple or any substances (9/39: 23.1%), of which three studies targeted alcohol and cannabis use, alcohol and tobacco use (*n* = 1), cannabis and tobacco (*n* = 1), alcohol, nicotine, and caffeine (*n* = 1), and any substance (*n* = 3).

Regarding the therapeutic or theoretical approaches used, most were feedback interventions (18/39: 46.1%), nearly all of which (13 of 18) provided comparisons with normative substance use among peers. As well, all of the 18 feedback interventions were for alcohol use and all were early interventions. Nine of the 39 interventions reported using a skills training approach, and 4 used cognitive bias modification. Importantly, only one of the 39 interventions employed a harm minimization approach. Definitions of these approaches can be found in Appendix 2. The names of the digital interventions, if provided by authors, were reported in Table 1; otherwise, a descriptive identifier was given.

### Spectrum of substance use treatment interventions and intended outcomes

**Fig. 2:**
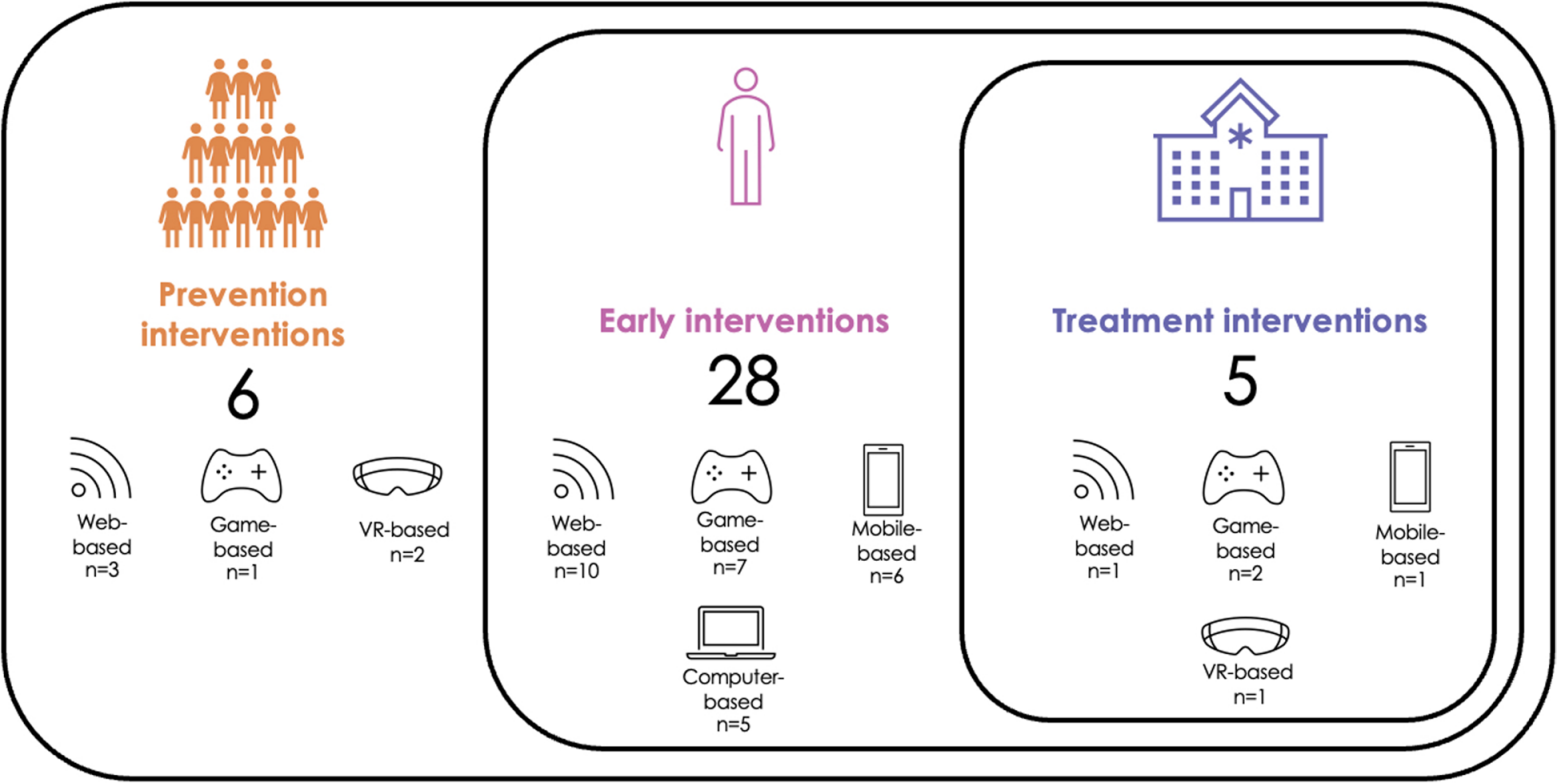
Distribution of interventions by level on the spectrum of substance use treatment

Regarding the three levels on the spectrum of substance use treatment [52], the vast majority in this review were early interventions (28/39: 71.8%), with nearly equal occurrences of prevention (6/39: 15.4%) and treatment (5/39: 12.8%) interventions. Figure 2 illustrates this distribution, including the types of digital technologies associated with each level (see Table 2). In terms of substances, most early interventions were geared towards alcohol use (20/28: 71%). No prevention interventions targeted alcohol or cannabis use only (Table 2).

**Table 2:** Distribution of digital interventions for substance use among young people by type of substance and intervention disposition (prevention/early intervention/treatment) *

	**Web-based**	**Game-based**	**Mobile-based**	**Computer/** **Tablet-based**	**Virtual Reality based**
**Prevention interventions**					
** Alcohol**					
** Tobacco/** **nicotine**					Invite Only (Weser, 2021 and Weser, 2021a) (101, 102) Virtual Reality Game in Smoking-Prevention Education (Guo, 2021) (103)
** Cannabis**					
** Opioids**	POP4Teens (Marsch, 2021) (73)				
** Any or multiple substances**	Any RealTeen (Schwinn, 2018 and Schwinn, 2019) (77, 78) Alcohol and cannabis Climate Schools Alcohol and Cannabis course, and other conditions (Champion, 2016; Newton, 2018 and Teesson, 2020) (74–76)	Tobacco and marijuana smokeSCREEN (Duncan, 2018) (86)			
**Early interventions**					
** Alcohol**	Alcooquizz (Bertholet, 2015) (62) OneTooMany (Davies, 2017) (63)^a^ The DEAL project (Deady, 2016) (64) Brief web-based intervention (Geisner, 2015) (65) Personalized Feedback Interventions (Miller, 2016) (66) Low response-based program (Schuckit, 2015) (67) Brief online alcohol use risk feedback intervention (Tuliao, 2019) (68) ALERTA ALCOHOL (Vargas-Martinez, 2019) (69)	Cheese Ninja Game (Boendermaker, 2015) (79)^a^ Ray’s Night Out (Hides, 2018) (81) Alcohol Alert (Jander, 2016) (82) Gamified PNF with co-presence (LaBrie, 2019) (83) CampusGANDR v2 (Earle, 2018) (80)	Approach Avoidance Task (Boendermaker, 2015) (79)^a^ D-ARIANNA (Carrà, 2015 Carrà, 2016) (89, 90) DrinksMeter (Davies, 2017) (63)^a^ SmarTrek (Kazemi, 2019) (91)	Computer delivered brief intervention (Ellis, 2017) (95) (95) Evaluative Conditioning Brief intervention (Tello, 2018) (96) Brief alcohol interventions (Walton, 2015) (97)	
** Tobacco/** **nicotine**	Put It Out Project (Vogel, 2020) (71)	HitnRun (Scholten, 2019) (84)			
** Cannabis**	Online personalized feedback intervention (Walukevich-Dienst, 2019) (72)			Computerized Approach Avoidance Training (Jacobus, 2018 and Karoly, 2019) (98, 99)	
**Opioids/Ketamine**					
** Any or mixed substances**		Alcohol, nicotine, caffeine Tetris (Skorka-Brown, 2015) (88)	Alcohol and Cannabis MiSARA (Coughlin, 2021) (94) Alcohol and Tobacco MobileCoachTobacco + (Haug, 2017) (93)	Alcohol or cannabis Computer-Based Substance Use Screening and Brief Behavioral Counseling (Knight, 2019) (100)	
**Treatment intervention**					
** Alcohol**					
** Cannabis**					
** Opioids/** **Ketamine**		Recovery Warrior (Abroms, 2015) (85)			Virtual reality-based vocational training system (Man, 2020) (104)
** Tobacco/** **nicotine**	Let’s Talk about Smoking (Brunette, 2018) (70)				
** Any or mixed substances**		Any Arise (Sanchez, 2015) (87)	Any ACHESS (Dennis, 2015) (92)		

^a^Multiple entries allowed to account for more than one digital technology intervention per study

**Fig. 3:**
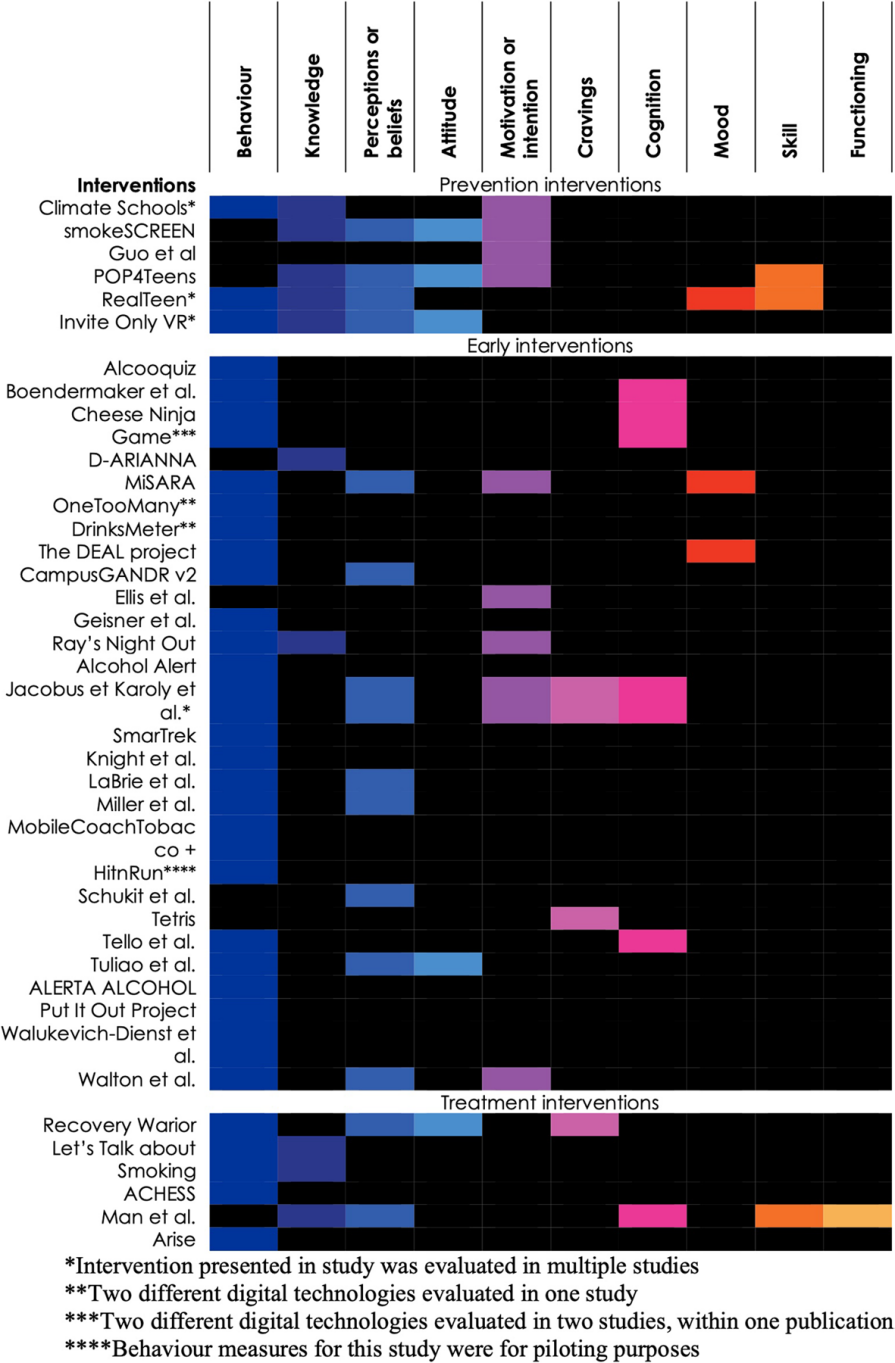
Intended outcomes of interventions stratified by level on the spectrum of treatment interventions

Figure 3 organizes the 39 interventions according to the three-level treatment spectrum (prevention, early intervention, and treatment) and maps the designated outcomes for each intervention. The most common designated outcome was a change in behaviour (33/39). Most early interventions (24/28) and nearly all treatment interventions (4/5) targeted behaviour change, compared with only half of studies using prevention interventions (3/6). Prevention interventions more often designated knowledge, perceptions or beliefs, attitudes, and intention to use substances as the intended outcomes.

### The nature of digital technologies in substance use interventions for youth

To address the main research question on the nature of digital technologies used in substance use interventions for young people, this section brings together the data on the five types of digital interventions identified in the review, describing which substances they targeted, the theoretical or therapeutic approaches used and level on the spectrum of treatment interventions.

#### Web-based interventions

Web-based interventions were the most commonly used technology (14/39), with most (8/14) targeting alcohol use: *Alcooquiz* [61], *One Too Many* [66], the *DEAL project* [67], the Geisner et al. intervention.[68], Miller et al. intervention [70], Schuckit et al. intervention [71], Tuliao et al. intervention [74] and *ALERTA ALCOHOL* [75]. All 8 interventions for alcohol use were early interventions, mainly geared to young adults, and all, except the *DEAL project*, provided users with feedback on their alcohol use (e.g., risks, consequences) [61, 66, 68, 70, 71, 74, 75]. Two web-based interventions targeted tobacco use (2/14): *Let’s talk about smoking* [62], and the *Put it Out Project* [76]. *Let’s talk about smoking* was a treatment intervention consisting of a brief intervention and motivational decision support [62], while the *Put it out Project* was an early intervention, based on US clinical guidelines and trans-theoretical models of behaviour change [76]. The Walukevich-Dienst [77] intervention, an early intervention with screening and personalized feedback, was the only web-based intervention for cannabis. Another, *POP4Teens,* was a web-based intervention for opioid use prevention based on psychoeducation, social influence, and skills training [69]. Other web-based interventions (2/14) targeted multiple, or any, substances: the *Climate School courses* [63–65] and *RealTeen*, tested in two studies by Schwinn et al. [72, 73]. The *Climate Schools courses* were a prevention intervention with an in-person component for alcohol and cannabis that used a social influence and harm-minimization approach [63–65]. *RealTeen* targeted prevention of any substance use and was based on psychoeducation, skills training, goal setting, social learning theory, and a resiliency framework [72, 73].

#### Game-based interventions

Game-based interventions, the second most common type of digital technology (10/39), mainly targeted alcohol use (5/10) and were all early interventions. They included the Boendermaker et al. intervention [79], *Campus GANDR v2* [81], *Ray’s Night Out* [82], *Alcohol Alert* [83], and the Labrie et al. intervention [84]. These interventions encompassed a range of theoretical approaches: cognitive bias modification [79], I-Change model and personalized feedback [83], motivational interviewing, from the Information-Motivation-Behavioural 2 skills health behaviour model and social learning theory [82], personalized normative feedback [81, 84] and self-determination theory [81]. *HitnRun* was the only game-based intervention for tobacco use, an early intervention based on peer contagion, that integrated principles of Go/No-Go training [86]. *Recovery Warrior* [78], a treatment intervention based on Social Cognitive Theory, Repetitive priming, and Reinforcement Theory of Motivation was the single game-based intervention targeting opioid use. Finally, *Smoke*-SCREEN [80], *Arise* [104] and *Tetris* [87] were the three (of 10) game-based interventions targeting multiple or any substances. *Smoke*-SCREEN targeted cannabis and tobacco prevention with behavioural skills development [80]; *Arise* for any substance and based on coping skills training [104]; while *Tetris* was an early intervention for alcohol, nicotine, and caffeine using elaborated intrusion theory and visual interference [87].

#### Mobile-based interventions

Seven of the 39 identified interventions were mobile interventions, most (4/7) targeting alcohol use. They included the Boendermaker et al. intervention [79], D-ARIANNA, tested in two studies by Carra et al. [88, 89], *Drinks Meter* [66], and *SmarTrek* [93] and all were early interventions for alcohol use. Boendermaker et al. used approach-avoidance training (cognitive bias modification) [79], while other interventions for alcohol included a feedback component. *SmarTrek* used motivational interviewing with ecological momentary interventions and personalized feedback [93]. *D-ARIANNA* did not specify a theoretical model but resembled a brief intervention with personalized feedback [88, 89]. *Drinks Meter* used personalized normative feedback based on psychoeducation [66]. The remaining mobile interventions (3/7) targeted multiple or any substances: *ACHESS* [91], *MobileCoach Tobacco* + [92], and *MiSARA* [90]. *ACHESS* was a treatment intervention for general substance use among adolescents at discharge from residential treatment, using ecological momentary intervention for support [91]. *MobileCoach Tobacco* + and *MiSARA* were early interventions. *MobileCoach Tobacco* + was for alcohol and tobacco, based on the Health Action Process Approach (HAPA), that took in a social norms approach, normative feedback, and social cognitive theory [92]. *MiSARA* was a support-based intervention for alcohol and cannabis use, based on personalized feedback, motivational interviewing, mindfulness, and behavioral activation [90].

#### Computer or Tablet-based interventions

Five of the 39 interventions were computer- or tablet-based. Most (3/5) targeted alcohol use: the Ellis et al. [94], Tello et al. [98], and Walton et al. [99] interventions. All were early interventions. Ellis et al. and Walton et al. included a brief feedback component and motivational interviewing [94, 99], while the Walton et al. intervention drew upon cognitive behavioural treatment and self-determination theory [99]. The Tello et al. alcohol intervention used cognitive bias modification [98]. The remaining two computer-based interventions included the unnamed intervention using Cannabis Approach Avoidance Training (CAAT), tested in two studies by Jacobus et al. and Karoly et al. [95, 96], which was the only intervention for cannabis, and an early intervention. The Knight et al. intervention [97] was an early intervention for multiple substances (alcohol or cannabis). Knight et al. employed motivational interviewing, psychoeducation, and a brief feedback intervention.

#### Virtual reality interventions

Virtual reality was the technology least employed among interventions in the review (3/39). Two interventions targeted tobacco/nicotine use and were both prevention-oriented: *Invite only VR*, tested in two studies by Weser, et al. [102, 103] and the Guo et al. intervention [100]. *Invite Only VR* targeted e-cigarette use and derived from behaviour change theories, the theory of planned behaviour and social cognitive theory [102, 103], while the Guo et al. tobacco prevention intervention was based on Keller’s ARCS (attention, relevance, confidence, and satisfaction) motivation model [100]. The virtual reality-based intervention by Man et al. targeted ketamine use [101]. This was a treatment intervention focused on cognitive problems in young adults using ketamine from a substance use clinic as well as residential and rehabilitation programs. This intervention involved training in cognitive and vocational skills.

## Discussion

As barriers to mental health and addiction services intensified during the Covid-19 pandemic, this rapid review was undertaken to provide an overview of studies on digital interventions for substance use among young people. The final review included 43 studies in total published between 2015 and 2021, describing 39 different interventions. We extracted and compiled data from these studies according to digital technologies used, substances targeted, the underlying theoretical or therapeutic models informing the interventions, and intended outcomes. The interventions were then organized according to treatment level (prevention, early intervention, treatment) following the spectrum of interventions framework developed by Stockings et al. Overall, this mapping of interventions reveals recent efforts in the addictions field to meet the needs of young people, particularly through digital education and interventions targeting some substances more than others. As well, early intervention programs reflected increasing personalization and interactivity, yet remained short on skills training. While many interventions recognized the harms of substance use, interventions based on an overall harm reduction approach were conspicuously absent in this review.

### Level on the spectrum of interventions and intended outcomes

For all types of technologies except virtual reality, the great majority of interventions (72%) were early interventions, outnumbering prevention, and treatment interventions at 14% each. The relative lack of treatment level interventions was interesting, as most of these interventions targeted youth in residential or outpatient treatment for serious addiction (e.g., opioids, ketamine). Yet, the paucity of prevention interventions was even more surprising, as substance use prevention is known to lower the odds of lifetime substance use [31]. As well, prevention interventions aimed to reduce interest in, or discourage substance use, as opposed to early interventions and treatment interventions dealing with youth at risk or already using drugs. Prevention programs tend to measure attitudes and knowledge rather than the incidence of substance use or harms [52]. Failing to report on behaviour outcomes diverts from evaluating the effects of prevention interventions on substance use, for example the cost-effectiveness of the intervention [52].

### Types of digital technologies

Most digital interventions in this study were web-based, yet web-based interventions for youth have low adherence or high drop-out rates [105]. Some studies suggest optimizing user engagement by developing gamified interventions to increase user attraction, participation, and entertainment [106–108]. As such, the second highest ranking for game-based interventions in this review was an encouraging finding, since games, particularly those offering a rich and interactive experience, have shown promising results in terms of user engagement [109].

Virtual reality-based interventions have also emerged as an effective way to provide substance use interventions, yet, according to findings in this review, virtual reality continues to receive little research attention. Virtual reality-based interventions have been used for their potential to simulate interactivity and motivate learning, and for their immersive properties [103, 110]. A recent review identified significant advantages related to virtual reality-based technologies for delivering educational content [111], while another review found that virtual reality may be effective for reducing substance use among adults [112]. However, the authors noted that more randomized controlled trials were still needed to establish efficacy.

### Substances targeted

Overall, the findings in this review related to substances targeted revealed a serious disjunction between the substances preferred by youth and those targeted by digital interventions. Alcohol use emerged as the substance most consistently targeted by all digital technologies, except virtual reality. The focus on alcohol use in half of the interventions was a hopeful sign, given that alcohol is the substance of choice for most youth [2]. However, much less research attention has been directed to other substances. For instance, tobacco and nicotine, the focus of only 12.8% of interventions in this review, is the second most prevalent substance used by youth [2]. Our review included only three interventions for e-cigarettes, despite the recent surge in e-cigarette use among young people internationally [4]. Moreover, our review identified only 2 interventions (6.6%) for cannabis, which was surprising given the increasing incidence of cannabis use, particularly in countries where cannabis has been legalized [6, 113], and research suggesting that people using marijuana are at high risk of graduating to illicit substances [9, 114].

The low number of digital interventions in the review for illicit drug use was unsurprising given the generally limited research on this type of intervention [51, 52, 115]. Only two treatment interventions and one prevention intervention targeted illicit drugs, among the interventions for mixed substances. As well, only one prevention digital intervention in the review targeted opioid use, despite expert opinion that prevention approaches are an underutilized strategy for mitigating the youth opioid crisis, given the low access to treatment for opioid use [116]. The limited attention to illicit drugs is concerning, since use of hard drugs in the early years, even when halted, is associated with premature decline in general health [117]. The increased risks of non-prescribed fentanyl and heroin use at 50% and 44%, respectively during the pandemic [26], underscore the urgency of developing digital and other treatment interventions for opioid use.

Interventions targeting any substance, or multiple substances, accounted for roughly one fourth of interventions in the review, another positive result given the prevalent use of combined substances and the reported increase in poly-substance use [118]. However, this review identified very few prevention interventions for either alcohol and cannabis or tobacco and cannabis. Given the high co-occurring use of alcohol, tobacco, and cannabis [25], associated in turn with the later initiation of illicit substances in young adulthood [21, 22, 119], the lack of prevention interventions for these substances is especially problematic. Given the mixed evidence on how substance use shifted during the pandemic, in relation to alcohol, tobacco or nicotine, and cannabis, firm conclusions have yet to be reached on current research needs regarding digital interventions for these substances.

### Theoretical and therapeutic approaches

For all types of technologies, except virtual reality, interventions in this review most often included a feedback component designed as an early intervention to halt escalation into problematic use. Feedback interventions provide information on personal substance use and associated risks. They may include a normative feedback component, based on a social norms approach, aiming to correct the tendency to overestimate substance use in others [115]. Social norms interventions are widely researched but, as Dempsey et al. observe [120], interventions using this approach may, or may not aim to change misperceptions. They further question the approach for lack of a robust theoretical model and the need for evaluation research that includes process evaluations and qualitative studies on patient experience with social norms interventions. The small effect of feedback interventions was consistent with a growing body of evidence suggesting that information provision for substance use tends to be ineffective in young people [26, 121]. Skills training seems more effective than information provision in prevention interventions, although there is still insufficient evidence for early interventions [122]. As such, approaches such as skills training, underutilized in the digital interventions identified in this review, may merit some further exploration. Virtual reality-based interventions, used for skills training in two of the three interventions cited in this review, may be particularly well suited for practicing skills [101].

Concerning harm reduction, only one intervention, the Climate Schools course [63–65], a web-based intervention for alcohol and cannabis prevention, used an explicit harm reduction approach that showed promising results for alcohol and cannabis knowledge, and for alcohol consumption and intended use. Harm reduction is an alternative to traditional abstinence-based treatment approaches that create barriers to treatment for young people who continue to use drugs [123, 124]. Harm reduction principles also promote more responsive and non-stigmatizing services by recognizing the realities of poverty, racism, social isolation, past trauma, sex-based discrimination, and other social inequities affecting individual vulnerability and the capacity to effectively deal with drug-related harms [125, 126]. Harm reduction approaches have been used successfully in early intervention programs to change attitudes through education [127, 128], and are also endorsed by professionals as an essential treatment approach for young people using opioids and other illicit drugs [123, 124, 129], who are known to prefer harm reduction to abstinence and sometimes devise their own harm reduction strategies [130, 131].

With stakeholders calling for a paradigm shift in the response to youth substance use [24], new avenues for the future evaluation of digital interventions may be considered. One possibility could involve the development of interventions using a harm reduction framework adapted to the socio-cultural realities and needs of young people using substances and delivered sequentially to support them along their substance use trajectories. Digital technologies with more interactive components may also be considered, game-based interventions for instance that have shown effectiveness in studies of mental health conditions [132, 133], and virtual reality interventions, given some evidence of its effectiveness in adult studies[109]. Moreover, the effectiveness of digital interventions may be enhanced by the participation of young people using substances as full partners in the design, testing and evaluation of digital interventions that concern them, as shown in video game implementation studies [134, 135]. Future research also needs to delve more deeply into various types of digital interventions in terms of their objectives and appropriateness for specific substances, while taking into account the characteristics of youth populations like age and substance use trajectories over time.

### Limitations

The findings of this rapid review included several limitations that should be addressed. As the review used a limited number of databases, the findings may underestimate the actual number of published interventions and give an imprecise account of research attention regarding the types of technologies, substances targeted, or therapeutic and theoretical approaches used. As only English articles were identified and analyzed, this review may have missed important interventions published in languages other than English. A critical appraisal of the included studies was not conducted, due to the heterogeneity of study methodologies and focus of the research team on the nature of interventions rather than their outcomes, and the desire to provide a comprehensive picture of digital interventions for substance use. Future reviews should assess the efficacy of digital interventions and technologies using critical appraisal and focusing on specific substance or digital interventions.

## Conclusions

Web-based interventions were the most common type of technology identified in this review, suggesting that digital interventions with more immersive components, such as game-based and virtual reality-based interventions were underutilized and may merit further exploration. The great majority of interventions also focused on alcohol use, revealing the need for more research attention to tobacco, cannabis, and the co-use of alcohol, tobacco, and cannabis, as well as illicit drugs. Given the predominance of early interventions in the review, the need for more prevention interventions for substance use becomes clear, especially interventions for generic substance use. Digital prevention interventions should also target substance use behaviours, as intended outcomes, to establish the efficacy of prevention-oriented interventions and improve available evidence. A prevention approach in interventions for opioid use may help mitigate the ongoing opioid crisis in North America. While most digital interventions included a feedback component, skills training approaches, underutilized in this review, may also prove effective.

## Supplementary Information

**Additional file 1.** AMSTAR – a measurement tool to assess the methodological quality of systematic reviews.

**Additional file 2: Appendix 1.** Definitions of digital intervention technologies (1-3).

**Additional file 3: Appendix 2.** Definitions of the most common theoretical or therapeutic approaches used indigital interventions for substance use among young people (4-11).

## Data Availability

Data screened and analysed in this rapid review are publicly available through the PubMed database (https://pubmed.ncbi.nlm.nih.gov/). Articles screened in this review may be requested from the corresponding author.
