# Correction: Digital interventions for substance use disorders in young people: rapid review

**DOI:** 10.1186/s13011-023-00529-y

**Published:** 2023-04-19

**Authors:** Marika Monarque, Judith Sabetti, Manuela Ferrari

**Affiliations:** 1 Douglas Mental Health University Institute, Montreal, QC Canada; 2 Department of Psychiatry, McGill University, Montreal, QC Canada

**Correction: Substance Abuse Treatment Prevention Policy 18, 13 (2023)**

**https://doi.org/10.1186/s13011-023-00518-1**

Following publication of the original article [1], the authors notified that changes identified and requested during the proofreading stage were not implemented correctly.

Authors’ affiliations should be as follows:

Marika Monarque1, Judith Sabetti1 and Manuela Ferrari1,2*

Also, there were several typo’s in the text and figures.

The citations in table 1 and table 2 linked to the wrong references.

The above typo and tables’ errors have been corrected, the changes are shown in supplementary file 1 (highlighted in yellow).

The original article 10.1186/s13011-023-00518-1 has been corrected.

## Supplementary Information

**Additional file 1.**  
