# Supplementary material for: Digital interventions for substance use disorders in young people: rapid review
Source: Subst Abuse Treat Prev Policy. 2023 Feb 17;18:13. doi: 10.1186/s13011-023-00518-1 (PMC9937742; doi:10.1186/s13011-023-00518-1)
Supplement: Supplementary file 2 — Additional file 2: Appendix 1. Definitions of digital intervention technologies (1-3). [file 13011_2023_518_MOESM2_ESM.docx]

**Appendix 1: Definitions of digital intervention technologies** (1-3)

| Term | Definition |
| --- | --- |
| Web-based intervention | Computerized program or service delivered through the internet (e.g., a website), and designed to create a positive change. |
| Video game-based intervention | Any game played on a digital device, encompassing a wide range of interfaces, including web-based programs and apps for mobile devices. These interventions include “serious” or “applied” games, which are games designed for a primary purpose other than entertainment. For this article, web-based, mobile-based, and computer-based interventions with a video game element were also classified in this category, as these interventions captured serious games or applied games designed for purposes other than entertainment. |
| Mobile-based intervention | A program delivered through a portable electronic or mobile device (e.g., smartphone). Examples include applications (‘apps’) or text/SMS-delivered interventions. |
| Computer-based or tablet-based intervention | A program delivered via computer or tablet. The intervention may be accessed on the internet or as an offline computerized program (e.g., CD-ROM, or installed software). For the purposes of this article, interventions were classified in this category if there was no mention of a video game or delivery through the internet. |
| Virtual reality-based intervention | A program delivered through virtual reality. Virtual reality allows individuals to enter into computer-generated simulations where they can interact with virtual surroundings. The surroundings can respond, in turn, to the actions of users in real time. |
