# Supplementary material for: Digital interventions for substance use disorders in young people: rapid review
Source: Subst Abuse Treat Prev Policy. 2023 Feb 17;18:13. doi: 10.1186/s13011-023-00518-1 (PMC9937742; doi:10.1186/s13011-023-00518-1)
Supplement: Supplementary file 3 — Additional file 3: Appendix 2. Definitions of the most common theoretical or therapeutic approaches used indigital interventions for substance use among young people (4-11). [file 13011_2023_518_MOESM3_ESM.docx]

**Appendix 2: Definitions of the most common theoretical or therapeutic approaches used in digital interventions for substance use among young people** (4-11)

| Feedback intervention | Feedback interventions for substance use provide information on personal substance use and associated risks. They may include personalized normative feedback, which provides information about social norms to correct the tendency to overestimate substance use in others. |
| --- | --- |
| Skills training approach | Skills training includes instruction, modeling, rehearsal or role-play, coaching and feedback to promote the acquisition of a skill. For example, this may include social skills such as refusing a substance from peers or problem-solving skills. |
| Cognitive bias modification | Cognitive bias modification interventions are designed to modify attention and interpretation biases related to substance use and involve repeated practice on cognitive tasks. |
| Harm reduction approach | Harm reduction is an alternative to traditional abstinence-based treatment approaches. Harm reduction principles promote more responsive and non-stigmatizing services by recognizing the realities of poverty, racism, social isolation, past trauma, sex-based discrimination, and other social inequities affecting individual vulnerability and capacity to effectively deal with drug-related harms. |

**Resources:**

1. Geraets CNW, van der Stouwe ECD, Pot-Kolder R, Veling W. Advances in immersive virtual reality interventions for mental disorders: A new reality? Current Opinion in Psychology. 2021;41:40-5.

2. Hollis C, Falconer CJ, Martin JL, Whittington C, Stockton S, Glazebrook C, et al. Annual Research Review: Digital health interventions for children and young people with mental health problems – a systematic and meta-review. Journal of Child Psychology and Psychiatry. 2017;58(4):474-503.

3. Ferreira-Brito F, Fialho M, Virgolino A, Neves I, Miranda AC, Sousa-Santos N, et al. Game-based interventions for neuropsychological assessment, training and rehabilitation: Which game-elements to use? A systematic review. Journal of Biomedical Informatics. 2019;98:103287.

4. Taylor JL, Johnson S, Cruz R, Gray JR, Schiff D, Bagley SM. Integrating Harm Reduction into Outpatient Opioid Use Disorder Treatment Settings : Harm Reduction in Outpatient Addiction Treatment. Journal of general internal medicine. 2021;36(12):3810-9.

5. Jenkins EK, Slemon A, Haines-Saah RJ. Developing harm reduction in the context of youth substance use: insights from a multi-site qualitative analysis of young people’s harm minimization strategies. Harm Reduction Journal. 2017;14(1):53.

6. Beard C. Cognitive bias modification for anxiety: current evidence and future directions. Expert Rev Neurother. 2011;11(2):299-311.

7. Elliott R, Malkin I, Gold J. Establishing safe injection facilities in Canada: legal and ethical issues. Can HIV AIDS Policy Law Rev. 2002;6(3):7-10.

8. Curry K. In Pursuit of Higher Pleasures: The Moral Value of Criminalizing Drug Users and the Utilitarian Case for Decriminalization [dissertation]. Ottawa (CA): Saint Paul University; 2019.

9. Dick S, Whelan E, Davoren MP, Dockray S, Heavin C, Linehan C, et al. A systematic review of the effectiveness of digital interventions for illicit substance misuse harm reduction in third-level students. BMC public health. 2019;19(1):1244-.

10. Boffo M, Zerhouni O, Gronau QF, van Beek RJJ, Nikolaou K, Marsman M, et al. Cognitive Bias Modification for Behavior Change in Alcohol and Smoking Addiction: Bayesian Meta-Analysis of Individual Participant Data. Neuropsychol Rev. 2019;29(1):52-78.

11. Platt JJ, Husband SD. An overview of problem-solving and social skills approaches in substance abuse treatment. Psychotherapy: Theory, Research, Practice, Training. 1993;30:276-83.
