# Supplementary material for: Correction: Digital interventions for substance use disorders in young people: rapid review
Source: Subst Abuse Treat Prev Policy. 2023 Apr 19;18:22. doi: 10.1186/s13011-023-00529-y (PMC10114306; doi:10.1186/s13011-023-00529-y)
Supplement: Supplementary file 1 — Additional file 1. [file 13011_2023_529_MOESM1_ESM.docx]

**Revisions on Proofs**: Digital interventions for substance use disorders in young people: rapid review

**Affiliations**:

Aff1 Douglas Mental Health University Institute, Perry E-3012, 6875 Boulevard LaSalle, Montreal, QC, Canada, H4H 1R3

Aff2 (same as Aff1)

Aff3 Department of Psychiatry, McGill University, Montreal, QC, Canada

**Abstract**:

AQ1: Caption for Figure 2 is: Figure 2: Distribution of interventions by level on the spectrum of substance use treatment.

AQ2: Yes, the author names are presented correctly.

- Methods
  - At line 1, add a comma after MEDLINE

AQ3: We have made corrections to the presentation of author affiliations as follows:

Aff1 Douglas Mental Health University Institute, Perry E-3012, 6875 Boulevard LaSalle, Montreal, QC, Canada, H4H 1R3

Aff2 (same as Aff1)

Aff3 Department of Psychiatry, McGill University, Montreal, QC, Canada

- Results
  - After ketamine (line 3), change (1) to (*n* = 1)

AQ4: We reviewed the entire article for the placement of sections and subsections, which coincide with our most recent submission with our responses to reviewer comments.

However, in the Results section, under the subsection “The nature of digital technologies in substance use interventions for youth”, the font on the subsequent group of third-order subsections (Web-based interventions, Game-based interventions, Mobile-based interventions, Computer or tablet-based interventions, Virtual reality interventions) is not uniform. The first heading “Web-based interventions” appears to be the correct size, while the others are too large (the same size as the second order subsections). Please verify and correct this.

AQ5: We have reviewed Tables 1 and 2, and find that the data are presented correctly, aside from many corrections made on Table 1, and changes in the reference numbers on Table 2 to coincide with the referencing done by the journal that changed the original sequence. We thank the editors for their considerable work re person-first language to improve Table 1.

**Table 1**:

| **Study** | **Substances** | **Intervention** | **Pop/N/age** | **Theory** | **Meth/Findings** |
| --- | --- | --- | --- | --- | --- |
| **Web-based interventions** | | | | | |
| Bertholet 2015 |  |  |  |  | **Add period** after vs (vs.) |
| Champion: change year (2015) to 2016 |  |  |  |  |  |
| Davies, 2017 |  | Change “One too many” to OneTooMany |  |  |  |
| Geisner 2015 |  |  | **Add -s** to BDI-II score (scores)  **Add -; age** after (SD: 1.34)**; age** range 18-24 |  |  |
| Schuckit, 2015 |  | Change “information” to “info” at lines 3,4 |  |  |  |
| Vargas-Martinez, 2019 |  | Change “information” to “info” at line 2 |  |  |  |
| Vogel, 2020 |  |  | **Add -s** to adult: young adults who smoke |  | Add hyphens: reduced abstinence at 3- and 6-months .. |
| Walukevich-Dienst  Missing year: add , 2019 |  |  |  |  |  |
| **Game-based interventions** | | | | | |
| Boendermaker, 2015 |  |  | Correct punct. And add age: (SD: 3.1); age range |  | **Add hyphen**:  Game-enhanced user… |
| Duncan, 2018 |  | Remove hyphen between *Smoke* and SCREEN: *smoke*SCREEN |  |  |  |
| Hides, 2017 Change to 2018 |  | Add -u to behavioral – line 4: behavioural; change “information” to “info” at line 3 |  |  |  |
| Jander, 2016 |  |  | **Add age**: age range 15-19 |  |  |
| LaBrie, 2019 |  | Change “information” to “info” at line 5 |  |  |  |
| Skorka-Brown, 2015 |  |  |  |  | Last line: change comma to semicolon:  ; consistent effect… |
| **Mobile-based interventions** | | | | | |
| Boendermaker, 2015 |  |  | **Make the n italic**: (*n* = 64)  Add “; age”: ; age range 18-35 |  |  |
| Carra, 2015 |  | Add “u” to behavior: behaviour at line 2 |  |  |  |
| Coughlin, 2021 |  |  |  |  | Change “1-month” to “1-mo." |
| Davies, 2017 |  | Remove the line break in description | Add “; age”: ; age range 18-30 |  |  |
| Haug, 2017 |  |  |  |  | Change significant to sig. and remove the ling break: No sig. group differences … |
| Kazemi, 2019 |  | Change 2 occurrences of “behavior” to “behaviour” | Italicize “n” in (*n* = 10) |  | Use abbreviation for “info”: “info was useful and had a positive effect…” |
| **Computer-based interventions** | | | | | |
| Ellis, 2017 |  | Change 2 occurrences of “behavior” (last 2 lines): “… at behaviour change. Tailored … drinking behaviours. |  |  |  |
| Jacobus, 2018 |  |  | Add ; age: 19; age range… |  |  |
| Karoly, 2019 |  |  | Typo (MRS). Change to “a 30-min. MRI scan |  | Change “significant” to “sig” – line 3. |
| Knight, 2019 |  | Change information to info at line 5 |  |  |  |
| Tello. 2018 |  |  |  |  | 2 instances of “behavior” to change to “behaviour” (lines 3, 4) |
| Walton, 2015 |  | For “Computer Brief intervention”, capitalize the I and add acronym (CBI) |  |  | Typo: Change “BI” to “CBI” at line 4 |
| **Virtual reality (VR)-based interventions** | | | | | |
| Man. 2018  Change the year 2018 to 2020 |  | Needs intervention name for “TAG”. Delete “a second program” and add “The Tutor-administered group (TAG) had similar …  Change spelling of “programme” to program – line 6 |  |  |  |
| Weser, 2021 |  | Change comma to period at line 4: “…e-cigarettes. Player uses … |  | Two occurrences of “behavior” – change to “behaviour” (lines 1,3) |  |
| Weser, 2021a |  |  |  |  | Remove comma in last sentence |
|  |  |  |  |  |  |
|  |  |  |  |  |  |
|  |  |  |  |  |  |
|  |  |  |  |  |  |
|  |  |  |  |  |  |
|  |  |  |  |  |  |

**Table 2**:

|  | **Web-based** | | **Game-based** | | **Mobile-based** | | **Computer/**  **Tablet-based** | | **Virtual Reality-based** |
| --- | --- | --- | --- | --- | --- | --- | --- | --- | --- |
| **PREVENTION INTERVENTIONS** | | | | | | | | | |
| **Alcohol** | | | | | | | | | |
| Tobacco/Nico | |  | |  | |  | |  | Change Invite Only to Invite Only VR, Weser, 2021 and Weser, 2021a (103, 104) |
| **Cannabis** | | | | | | | | | |
| Any or multiple substances | | Schwinn, 2018 and Schwinn, 2019 (73, 74);  Champion, 2016; Newton, 2018 and Teeson, 2020 (64-66) | | Italicize “smoke”: *smoke*SCREEN  Duncan, 2018 (81) | |  | |  |  |
| **EARLY INTERVENTION** | | | | | | | | | |
| Alcohol | | (Davies, 2017) (67)  Deady, 2016 (68)  Geisner, 2015 (69)  Miller, 2016 (71)  Schuckit, 2015 (72)  Tulaio, 2019 (75)  Vargas-Martinez (2019 (76) | | Boendermaker, 2015 (80)  Hides, 2018 (83)  Jander, 2016 (84)  LaBrie, 2019 (85)  Earle, 2018 (82) | | Boendermaker, 2015 (80)  Davies, 2017 (67)  Kazemi, 2019 (94) | | Ellis, 2017 (95) Remove repetition of (95)  Tello, 2018 (99)  Walton, 2015 (100) |  |
| Tobacco/  Nicotine | | Vogel, 2020 (77) | | Scholten, 2019 (87) | |  | |  |  |
| Cannabis | | Walukevish-Dienst, 2019 (78) | |  | |  | | Jacobus, 2018 and Karoly, 2019 (96, 97) |  |
| Opioids/ Ketamine | | | | | | | | | |
| Any or mixed substances | |  | | Skorka-Brown, 2015 OK | | Coughlin, 2021 (91)  Haug, 2017: Make a space after Coach: MobileCoach Tobacco + | | Knight, 2019 (98) |  |
| **TREATMENT INTERVENTION** | | | | | | | | | |
| Opioids/ Ketamine | |  | | Abroms, 2015 (79) | |  | |  | Man, 2020 (102) |
| Tobacco/nicotine | | Brunette, 2018 (63) | |  | |  | |  |  |
| Any or mixed substances | |  | | Sanchez, 2015 (86) | |  | |  |  |
|  | |  | |  | |  | |  |  |
|  | |  | |  | |  | |  |  |

- Supplementary Information

**INTRODUCTION**:

AQ6: The caption on Figure 2, which illustrates the numbers of prevention, early intervention, and treatment modalities in the study is missing. The caption should read: Figure 2: Distribution of interventions by level on the spectrum of substance use treatment.

METHODS

Research Questions

AQ7:

**Search Strategy**:

- At line 4, replace the quotation mark before OR “Smoking” (because it looks weird)

AQ8:

**Fig. 3:**

- Five typos:
  - Change smokeSCREEN to *smoke*SCREEN
  - Jacobus et Karoly et al – Change « et » to « & »
  - MobileCoach Tobacco (add space after Coach)
  - Change Schukit : Change to Schuckit
  - Change Recovery Warior to Warrior (2 r’s)

**The nature of digital technologies in substance use interventions for youth**:

- Web-based interventions:
  - Maybe add a reminder here that the heading “Web-based interventions” is the appropriate font size for all 5 subsections within this heading.
  - Change “*One Too Many*” to “*OneTooMany*”
- Game-based interventions:
  - Capitalize the “B” in LaBrie
  - Remove hyphen in Smoke-SCREEN and no capital “S”: *smoke*SCREEN.
  - Same correction in the following line (7): *smoke*SCREEN.

- Mobile-based interventions:
  - Carra (line 2) has an accent: Carrà

**DISCUSSION**:

- Last paragraph, line 4: change “its” to “their” “… and virtual reality interventions, given some evidence of their effectiveness in adult studies”
